# Understanding subjective well-being: perspectives from psychology and public health

**DOI:** 10.1186/s40985-020-00142-5

**Published:** 2020-11-19

**Authors:** Kirti V. Das, Carla Jones-Harrell, Yingling Fan, Anu Ramaswami, Ben Orlove, Nisha Botchwey

**Affiliations:** 1 Department of Civil and Environmental Engineering, Princeton University, E208 Engineering Quadrangle, Princeton, NJ 08544 USA; 2 Rollins School of Public Health, Emory University, 1518 Clifton Rd, Atlanta, GA 30322 USA; 3 Hubert H. Humphrey School of Public Affairs, University of Minnesota, 301 19th Avenue South, Minneapolis, MN 55455 USA; 4 School of International and Public Affairs, Columbia University, New York, NY 10027 USA; 5 College of Design, Georgia Tech, 245 4th Street, NW, Suite 204, Atlanta, GA 30332 USA

**Keywords:** Subjective well-being, Theories, Determinants and correlates, Interdisciplinary review, Public health, Psychology, Happiness, Life evaluation, Affective, Cognitive

## Abstract

**Background:**

Individual subjective well-being (SWB) is essential for creating and maintaining healthy, productive societies. The literature on SWB is vast and dispersed across multiple disciplines. However, few reviews have summarized the theoretical and empirical tenets of SWB literature across disciplinary boundaries.

**Methods:**

We cataloged and consolidated SWB-related theories and empirical evidence from the fields of psychology and public health using a combination of online catalogs of scholarly articles and online search engines to retrieve relevant articles. For both theories and determinants/correlates of SWB, PubMed, PsychINFO, and Google Scholar were used to obtain relevant articles. Articles for the review were screened for relevance, varied perspectives, journal impact, geographic location of study, and topicality. A core theme of SWB empirical literature was the identification of SWB determinants/correlates, and over 100 research articles were reviewed and summarized for this review.

**Results:**

We found that SWB theories can be classified into four groups: fulfillment and engagement theories, personal orientation theories, evaluative theories, and emotional theories. A critical analysis of the conflicts and overlaps between these theories reveals the lack of a coherent theoretical and methodological framework that would make empirical research systematically comparable. We found that determinants/correlates of SWB can be grouped into seven broad categories: basic demographics, socioeconomic status, health and functioning, personality, social support, religion and culture, and geography and infrastructure. However, these are rarely studied consistently or used to test theories.

**Conclusions:**

The lack of a clear, unifying theoretical basis for categorizing and comparing empirical studies can potentially be overcome using an operationalizable criterion that focuses on the dimension of SWB studied, measure of SWB used, design of the study, study population, and types of determinants and correlates. From our review of the empirical literature on SWB, we found that the seven categories of determinants/correlates identified may potentially be used to improve the link between theory and empirical research, and that the overlap in the determinant/correlates as they relate to multiple theory categories may enable us to test theories in unison. However, doing so in the future would require a conscious effort by researchers in several areas, which are discussed.

## Background

Well-being has long been considered key to the creation and maintenance of healthy, productive societies [1, 2]. To this end, many countries utilize objective proxies of well-being, such as income, literacy, and life expectancy, as well as subjective measures, such as how life is perceived and experienced by individuals [2]. This approach to measuring perceptions and life experiences has been characterized as *subjective well*-*being* (SWB). Diener [3], one of the leading scholars in SWB research, defines *SWB* as “a person feeling and thinking his or her life is desirable regardless of how others see it.” This definition highlights the *thinking* and *feeling* dimensions of SWB:

- *Feeling* refers to the emotional/affective dimension (EMO) of SWB, where a preponderance of positive emotion over negative emotion leads to higher SWB.
- *Thinking* refers to the evaluative/cognitive dimension (EVA) of SWB, where the evaluation of individuals’ lives in predominantly positive terms leads to higher SWB.

To avoid nomenclature confusion, in the following review, SWB refers to both evaluative and emotional SWB, EVA refers to the evaluative aspect, and EMO refers to the emotional aspect.

Diener’s [3] focus on the subjectively reported feeling and thinking states adheres to a hedonic view of well-being [4–6]. In contrast, the eudemonic view emphasizes the realization of a person’s potential [7, 8]. According to the eudemonic view, well-being is a normative construct regarded as the possession of certain desirable qualities. As Diener [3] points out, eudemonic well-being does not reflect “the actor’s subjective judgment, but the value framework of the researcher.” In this review, we chose not to focus on eudemonic well-being—an external assessment of whether someone is living a desirable or purposeful life—but rather on hedonic well-being—the individual’s own sense of his or her well-being, as designated by EVA and EMO.

The literature on SWB is vast and dispersed across multiple disciplines. SWB-related studies appear in traditional disciplines, such as philosophy, economics, and psychology, as well as in emerging fields, such as public health and human ecology. Previous literature reviews of SWB [9–11] tended to focus on a single discipline. Given that theoretical and empirical SWB studies are often scattered across disciplinary boundaries, reviews within a single discipline rarely consolidate theories and empirical evidence together for new insights into research directions. To address this gap, the current paper attempts to provide a consolidated review of SWB-related theoretical and empirical studies across the psychology and public health disciplines with the intent to develop a theoretical and methodological framework that would make empirical research comparable in a systematic way.

The focus on the disciplines of psychology and public health was selected largely because of the complementary analytical frameworks and methods of the two disciplines when it comes to understanding SWB, especially the emphasis of the former on individuals and of the latter on populations. The theoretical underpinning of SWB concepts have historically drawn on many disciplines, but it is generally acknowledged that psychology has been the most important contributor discipline for identifying theoretically relevant determinants of SWB and the mechanisms through which determinants influence SWB [12]. In contrast, SWB studies in the public health discipline have frequently been criticized for being atheoretical [13–16] and have primarily focused on empirically identifying relevant determinants and correlates of SWB. Over time, a more sophisticated and consolidated view of the psychological theories and the empirical public health research is needed for advancing both the theoretical development and empirical examination of SWB.

Based upon a consolidated review of the psychology and public health literature, we summarize and classify theoretical SWB studies into four major categories: fulfillment and engagement theories, personal orientation theories, evaluative theories, and emotional theories. We provide a critical analysis of the conflicts and overlaps between these theories. The analysis helps to reveal that the SWB literature lacks a coherent theoretical and methodological framework that would make empirical research comparable in a systematic way. Instead of trying to generate a coherent framework (that is currently theoretically and methodologically impossible) to inform the review of the empirical literature, we propose an operationalizable criterion that focuses on dimension of SWB studied, measure of SWB used, design of the study, study population, and types of determinants and correlates to organize and summarize the empirical literature.

We find that although many theories of SWB originate in psychology, empirical studies identifying SWB determinants and correlates are dispersed across psychology and public health research. Our review of the empirical literature revealed that psychology and public health studies focus on diverse sets of SWB determinants and correlates, ranging from demographics, personality, geography, and supportive relationships to health status [17, 18]. Based on our criterion for categorizing and comparing empirical studies, we find numerous challenges limiting comparability including inconsistencies in the dimension of SWB studied, the measures of SWB used, design of studies, the samples used to collect data, and the determinants and correlates included in studies. In addition, very few empirical studies closely follow SWB theories. Most of the empirical studies focus on examining how determinants or correlates influence EVA and EMO. Although theoretical studies indicate that SWB determinants and correlates may interact according to different contextual factors [19], very few empirical studies explore these interactions.

The findings from the review of empirical studies are not surprising and align with the findings from the review of the theories: there is a lack of congruence of basic categories of analysis, epistemological assumptions, and a number of competing and overlapping theories about SWB and its determinants, within which there are potentially contradictory claims or incommensurable elements. Given the difficulties in using theories to generate comparable empirical studies, we make an argument toward the end of the paper that an empirical summary of determinants/correlates of SWB can potentially be used to improve the link between theory and empirical research and that the overlap in the determinant/correlates as they relate to our four theory categories may enable us to conduct theoretical testing across theories in unison. However, to do so in the future requires a conscious effort by researchers in several areas which are discussed.

## Methodology

We used a combination of scholarly article online catalogs and online search engines to retrieve relevant articles by discipline. The search was conducted in two stages, first for SWB theories and then for determinants. PubMed, PsychINFO, and Google Scholar were used to obtain relevant articles. Articles were first screened by title, abstract, and keywords. For SWB theories, the inclusion criteria were phrases such as “subjective well-being” and “subjective well-being theories.” For determinants, the inclusion criteria were phrases such as “subjective well-being predictors” and “subjective well-being determinants.” For determinants, a second query was crafted based on keywords commonly found among the results from the first search. For example, “subjective well-being determinants” returned many articles on age and SWB, so a follow-up search was conducted using the keywords “age and subjective well-being.” Abstracts were reviewed for relevance, varied perspectives, avoidance of overlap, journal impact, geographic location of study, and topical area. This process led to the selection of 35 (of 68 retrieved) articles related to theories and 105 (of 158 retrieved) articles related to determinants and correlates, all of which were reviewed in detail. The articles selected were published between 1965 and 2018. It is important to distinguish our review from a systematic review of SWB theories and empirical literature. The intent of this review is to identify areas of congruence and incongruence in the theoretical and empirical bodies of literature that can inform the future development of a framework that would make empirical research comparable and aid systematic reviews in the future.

## Results

### The theoretical foundations of SWB

We found that SWB theories tend to originate from psychology and focus on the mechanisms through which individual SWB is affected by internal (personal) and external (social) factors.

**Fig. 1:**
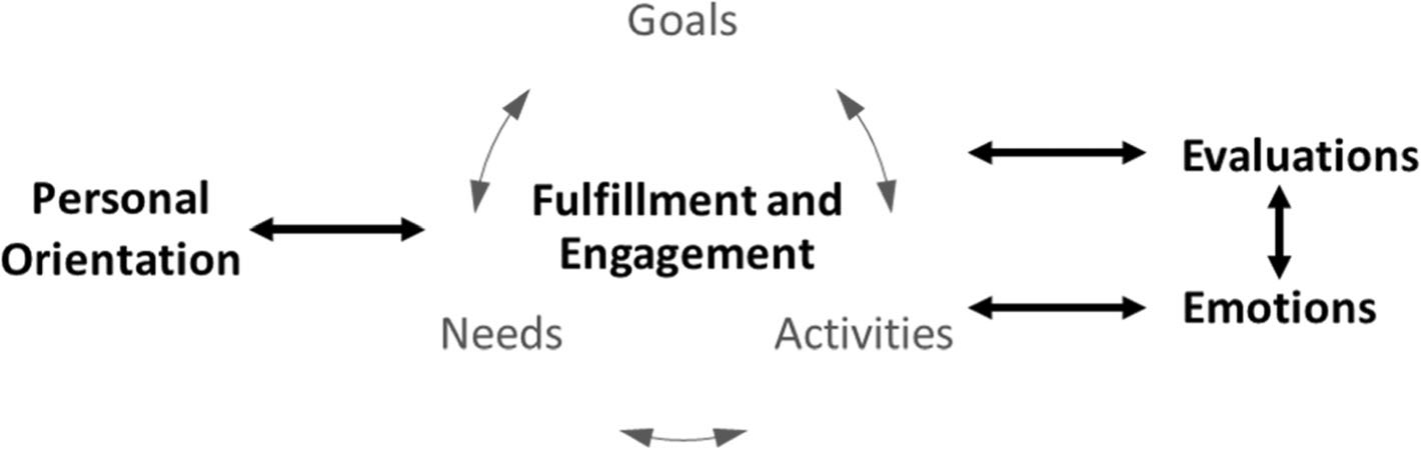
Associations between theories of SWB

We group SWB-related theories into four major categories: fulfillment and engagement theories, personal orientation theories, evaluative theories, and emotional theories. Figure 1 summarizes the intermediate constructs and mechanisms that underlie the causes and effects of SWB among the theories. Our categorization of theories and the associations between them (as shown in Fig. 1) is a preliminary attempt at integration or synthesis of theories to understand them in unison rather than in isolation. As researchers work toward the creation of comprehensive testable propositions which can be linked to potential determinants and correlates of SWB in existing literature, we expect this figure to evolve significantly.

According to these theories, any factors that affect personal orientation, goals, needs, activities, evaluations, or emotions are determinants of SWB. Each category of theories focuses on a different mechanism in which SWB is affected:

- Fulfillment and engagement theories focus on explaining the influences of goals, needs, and activities on SWB.
- Personal orientation theories focus on explaining the influence of temperament on SWB by dynamically affecting the process of fulfillment and engagement as well as how the dynamic process leads to the readjustment of personal orientation.
- Evaluative theories focus on how personal evaluations of life (i.e., the cognitive aspect of SWB) are interconnected with the process of fulfillment and emotions.
- Emotion theories focus on how experiences of emotions (i.e., the affective aspect of SWB) are interconnected with the process of fulfillment, engagement, and evaluations.

#### Fulfillment and engagement theories

The first subset, fulfillment and engagement theories, are telic theories. These theories contend that SWB increases when certain needs or goals are met. Such theories are highlighted in the works of Maslow [20] and Wilson [21]. As defined by Wilson [21], the “satisfaction of needs causes happiness, and conversely, the persistence of unfulfilled needs causes unhappiness.” Telic theories can be need-focused or goal-focused. Need-based theories deal with certain inborn or learned needs that a person may or may not be aware of but which, once met, lead to SWB. Goal-based theories focus on specific desires that a person is aware of and the actions taken to fulfill them; the fulfillment of these desires ultimately leads to SWB [22].

Another subset of fulfillment and engagement theories focuses on how emotions can be both determinants and outcomes of SWB. These theories emphasize that pleasure and pain are often associated with need and goal fulfillment. Pleasure and pain are thereby connected concepts that rely on an individual’s psychological engagement with the goal or need they wish to fulfill [23]. The greater the psychological engagement, the greater the pleasure when it is achieved, or dissatisfaction when it is not. While most of these theories have to do with psychological gains and losses, including positive and negative emotions through activities to achieve needs and goals, some theories extend gains and losses to include physical resources. For example, physical deprivation has been theorized as a prerequisite to psychological engagement with a need or goal [3, 24]. Solomon’s [25] opponent process theory postulates that the loss of something good causes pain, while the loss of something bad causes pleasure.

The final subset of fulfillment and engagement theories focuses not on an outcome, such as evaluations and emotions resulting from an occurrence, but on the process of the occurrence itself. With roots tracing as far back as Aristotle, such theories suggest that SWB is a byproduct of human activity wherein deeper engagement in an activity performed satisfactorily that fits an individual’s skillset leads to higher SWB. Csikszentmihalyi’s [26] theory of flow purports that activities are most pleasurable when the challenge of activity demands deep engagement and matches an individual’s skills. Concentrating on gaining happiness and SWB, in and of oneself, can be self-defeating; scholars suggest that focusing on goals- or needs-driven activities leads to SWB as an unintended byproduct [27].

While fulfillment and engagement theories are frequently mentioned in the literature, such mentions are often ambiguous; few have been systematically formulated or empirically tested. For telic theories, several universal human needs, including efficacy, self-approval, and understanding, have been proposed and contested [28]. The lack of clarity in defining universal needs or goals, however, makes these theories difficult to test. Nonetheless, it is worth noting that fulfillment and engagement theories could be used to bring together two related but often disparate conceptualizations of SWB: eudemonic and hedonic. As mentioned earlier, the hedonic view of SWB focuses on subjective feelings and thinking states, while the eudemonic view emphasizes the realization of a person’s potential and the possession of certain desirable qualities. There is a long-standing debate within psychology about the independence of eudemonic and hedonic SWB. Fulfillment and engagement theories uncover the important overlaps and convergences between these two conceptualizations.

#### Personal orientation theories

Although demographic variables, such as health, income, educational background, and marital status, have been widely considered as both determinants and proxies for needs, goals, and activities, an exploration of these demographic variables demonstrates that they only account for small variations in SWB [29, 30]. The search for better determinants has led researchers to delve deeper into personal temperament and how it may impact SWB. Many of these theories suggest that SWB is primarily determined by our inborn predispositions [18, 31]. Personal orientation theories propose that while SWB may be associated with need or goal fulfillment, personality and personality–environment fit can be just as important.

Headey and Wearing’s [32] dynamic equilibrium model suggests that everyone has a unique baseline level of SWB, primarily determined by their personal orientation. Circumstances may result in swinging above or below this baseline, but all eventually return to it. Similarly, set-point theory also suggests that the effects of life events on SWB are only temporary and eventually regress to a baseline (default) determined by personality and genetics [31, 33].

Others have theorized that personality not only influences the likelihood of the events encountered (e.g., marriage or employment) but also causes people to react to certain events emotionally in ways that impact their SWB. Based on research focused on the Big Five personality traits of extroversion, agreeableness, openness, conscientiousness, and neuroticism [34], the two most prominent personality traits in relation to SWB are extroversion and neuroticism [35, 36]. Neuroticism has been linked to a higher incidence of negative emotions, such as anxiety, fear, frustration, anger, loneliness, and depression. Extroversion has been linked to being more social, gregarious, cheerful, and excitement-seeking. Resting on Gray’s [37] theory of personality, Tellegen [38] and Rusting and Larsen [39] theorized that extraverts are more reactive to pleasant emotional stimuli than are introverts, and neurotic individuals are more reactive to unpleasant emotional stimuli than are stable individuals. The role of personal orientation is critical to SWB, as it may determine one’s baseline SWB, activity engagement, the events or situations faced in life, and how they are experienced.

#### Evaluative theories

Evaluative theories view SWB as a mental comparison between an individual’s life, conditions, or circumstances with a specific objective or subjective standard. Personal conditions exceeding this standard result in higher SWB, and vice versa. These comparisons and their related levels of SWB can be conscious, as in life evaluation, or unconscious, as in emotional processes. As such, evaluative theories consider evaluations to be both key outcomes and determinants of SWB.

Evaluative theories can be further divided into two sub-groups based upon how the standard used for comparison is formulated. In social construction theory, peers are often used as the standard for comparison. If an individual thinks of himself or herself better off than others, he or she will have higher SWB [22, 40, 41]. In adaptation and range–frequency theory, an individual’s past is often used to set this standard [42–44]. If his or her current life exceeds this standard, that person will have higher SWB. Others, such as Meadow et al. [45], focus on income and suggest a combined effect where the standard is invariably a combination of one’s past and the situation of others. Social construction theory also suggests that this standard is an individual’s assessment of what life ought to be [46]. This standard views SWB as a shared societal or collective notion such as “beauty” or “fairness” that frames how people make social comparisons. Under social construction theory, SWB is the gap between perceptions of what life is and ideas of how life should be [47]. The standard for comparison is not experience-, event-, or feeling-driven; instead, it is driven by the notion of what SWB is.

Adaptation theory deserves a special mention, as it relates to an individual’s formulation of standards. The theory postulates that there is an individual baseline for SWB that moves up or down based on one’s life conditions, situations, and experiences. In this way, it differs from both the dynamic equilibrium model and set-point theory mentioned above, which focus on personality as the driving factor of the baseline. Adaptation theory suggests that when events first occur, they can have a positive or negative impact on SWB. Over time, however, a person adapts to such events, and their impact on SWB lessens [42, 48]. For example, if events in one’s personal life are above the current standard, this will improve SWB, but as these positive events continue, the person adapts to them and their standard will rise until these positive events become the new standard.

#### Emotion theories

Emotion theories focus on how positive and negative emotions are not only outcomes but also intermediate and direct determinants of SWB. Although this set of theories overlaps somewhat with fulfillment and engagement theories that emphasize the relation of pleasure and pain to need and goal fulfillment, emotion theories represent important extensions that demonstrate additional mechanisms through which emotions affect activities and reinforce SWB.

Fredrickson’s [49, 50] broaden-and-build theory of positive emotions is one of these extensions. The theory explores the form and function of a subset of positive emotions that include joy, interest, contentment, and love [50]. The theory posits that positive emotions broaden an individual’s momentary thought–action repertoire. Frederickson [50] contended that joy sparks an urge to play, while interest sparks an urge to explore. The second key element of this theory proposes the consequentiality of positive emotions, suggesting that these emotions build on an individual’s enduring personal resources, ranging from physical and intellectual to social and psychological resources [49]. These newly built resources and newly broadened activities further reinforce SWB and form a positive feedback loop between long-term SWB and short-term emotions.

Other theories look at affective experiences through the lens of associative memory networks. Positive experiences trigger positive memories, which contribute to higher SWB. Bower [51] has shown that people recall memories that are affectively congruent with their current emotional state. Based on frequent affective experiences, individuals are affectively conditioned, and this conditioning can be extremely resistant to extinction. Thus, happy people might be those who have had positive affective experiences associated with frequent positive stimuli. Zajonc [52] contends that affective reactions occur independently of, and more rapidly than, the cognitive evaluation of stimuli and are compatible with a conditioning approach to happiness.

#### Conflicts and overlaps between theories

Our review of theories revealed three important issues. First, we found that in many cases, there is a lack of conceptual development of theories to measurable and testable frameworks. For example, when it came to fulfillment and engagement theories, a lack of systematic formulation has left several questions unanswered. Are there universal sets of needs and goals that can best tested across populations and compared? How do an individuals’ goals and needs vary based on factors such as age, economic status, family structure, social structure, etc.? Are people aware of their needs and goals? Can these needs and goals be measured directly or do they require proxy measures? Similarly, with personal orientation theories, there is little agreement on what aspects of the personality should be tested in relation to SWB with some studies focusing on the Big Five personality traits and others focusing on more nuanced traits such as optimism and self-efficacy.

Second, we find that theories can be both competing and overlapping with few attempts to synthesize theoretical/conceptual frameworks. This could potentially result in empirical studies related to SWB following specific theoretical directions and limiting comparability and synthesis across this area of research. This seems counterintuitive to calls from leading researchers in the field to test multiple theories and their propositions simultaneously to gain a more cohesive view on the structure of SWB [3]. For example, there are gaps in understanding how personal orientation, standards of comparison, and ones’ culture influence goal and need formation and the level of SWB associated with completing or accomplishing them. Little is known about how emotions that build an individual’s enduring personal resources, ranging from the physical and intellectual to social and psychological, impact ones’ ability to formulate and meet goals and needs. Personal orientation theories rarely mention the potential impacts of social and economic resources, culture, and access to activities (to meet needs and goals) in influencing SWB. It would be beneficial to understand if the influence of personality on SWB is moderated by the environment (social conditions, economic conditions, or other life circumstances) one lives in or if the extent of this influence depends on the ability to conduct trait congruent behaviors and activities. Similarly, for evaluative theories, it would be important to understand if the standards of comparison people use to assess their SWB are absolute or relative in terms of where a person stands in life (in terms of age, economic status, family structure, social structure, etc.) or if a persons’ tendency to compare upwards or downwards (based on their life situation) depends on their personal orientation. Finally, for emotional theories, it would be important to know how personal orientation influences thought-action repertoire and building of resources and does ones’ ability to conduct activities congruent with resource building influence how emotions influence SWB.

Finally, while not the focus of this research, it is important to point out that eudemonic theories can be argued to overlap with almost all theories mentioned here, a common problem with a priori theories. For example, the broaden and build theory looks at the role of emotion in building resources and resilience. These are related to both emotions and eudemonia. Similarly, theories such as Maslow’s hierarchy of needs (and its SWB iterations) identify specific needs that one must fulfill for higher SWB which relate to both fulfillment theories and eudemonic theories.

In summary, we used a few examples to illustrate plausible connections between theories that need to be explored and questions that cannot be answered unless these theories are tested in unison. One of the key challenges in accomplishing this is that there is a lack of a clear basis for categorizing and comparing empirical studies for systematic reviews that can help fill these gaps. In the next section, instead of trying to generate a coherent framework (that is currently theoretically and methodologically impossible) to inform the review of the empirical literature, we propose an operationalizable criterion that focuses on dimension of SWB studied, measure of SWB used, design of the study, study population, and types of determinants and correlates to organize and summarize the empirical literature.

### Determinants and correlates of SWB

SWB theories have led to the identification of potential determinants/correlates and their empirical testing. Factors potentially influencing personal orientation, fulfillment and engagement, evaluations, and emotions are considered determinants/correlates of SWB. We found that, despite the extensive theoretical studies, very few empirical studies closely follow SWB theories. Most of the empirical studies focus on examining how determinants and correlates influence EVA and EMO.

Unlike SWB theories grounded in psychology, empirical studies are found in both public health and psychology. However, the two disciplines have different empirical aims and interests. Public health studies tended to highlight how specific health conditions affect SWB, and psychology studies tended to highlight how personality traits affect SWB.

Based on our review and consideration of all identified determinants/correlates, we found that they fall into seven broad categories:

1. *Basic demographics:* gender, age, and race/ethnicity
2. *Socioeconomic status* (SES): income, education, employment, family structure, and immigration status
3. *Health and functioning*: general or self-reported health, diseases, mental and physical disability, obesity, sleep deprivation, and physical activity
4. *Personality*: the Big Five personality traits and nuanced traits, such as self-efficacy, optimism, and self-esteem
5. *Social support*: the number of contacts, quality of contacts, friends, family, family satisfaction, social satisfaction, and discrimination
6. *Religion and culture*: conceptualization of SWB, formulation of comparison standards, religiosity, and visits to houses of worship (mosque, temple, synagogue, etc.)
7. *Geography and infrastructure*: conditions at various levels of disaggregation, including nation, region, community (city, town, or parish), neighborhood, and home, and access to infrastructures, such as food, water, sanitation, transportation, greenery, leisure, and ecosystems

Next, we formulated a criterion to group existing empirical literature to assess comparability that could aid in conducting systematic reviews in the future. This involved looking at studies based on:

1. Dimension of SWB studied: Conceptualization of SWB varies across the literature. While there is consensus in literature about the existence and independence of EVA and EMO dimensions of SWB, most studies focus on one or the other. If the dimension of SWB being measured across studies is not consistent, it limits their comparability.
2. Measurement of SWB used: There is little agreement on the structure of SWB and therefore the scales used to measure it vary significantly. The use of numerous tools with varying components to measure SWB and its dimensions could impact comparability of studies.
3. Design of study: Most empirical studies aim to identify determinants of SWB; therefore, the use of longitudinal or experimental study designs would be best suited to tease out causal relationships. The use of cross-sectional designs would at best, be able to identify correlates of SWB. Hence, studies with varying temporal designs would hinder comparability.
4. Study population: If we hope to generalize from or compare studies, it is very important that the samples used are comparable. In addition, if the conceptualization of SWB and its structure varies across groups of people based on culture, religion, etc., even if SWB is measured using the same measurement tool, the results may not be comparable.
5. Determinants or correlates used: To be able to systematically compare studies, they would need to test similar determinants or correlates of SWB. For example, in terms of comparing potential effect sizes, consistent specifications of models are crucial. Using consistent model specification across different samples would also be critical to identifying any universal drivers of SWB.

**Fig. 2:**
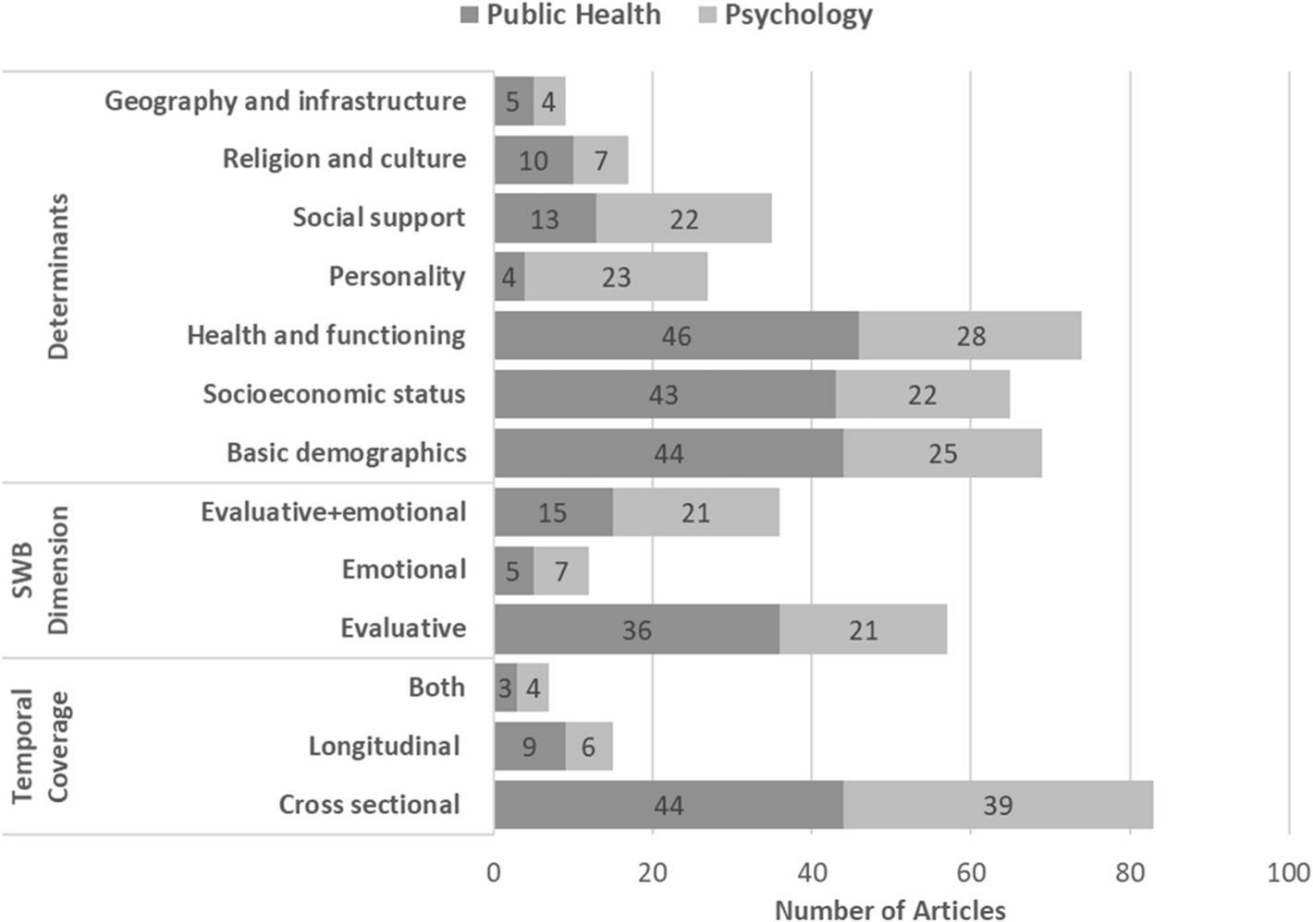
Distribution of reviewed studies (published between 1965 and 2018) by temporal coverage, SWB dimension studied, and determinants and correlates

**Fig. 3:**
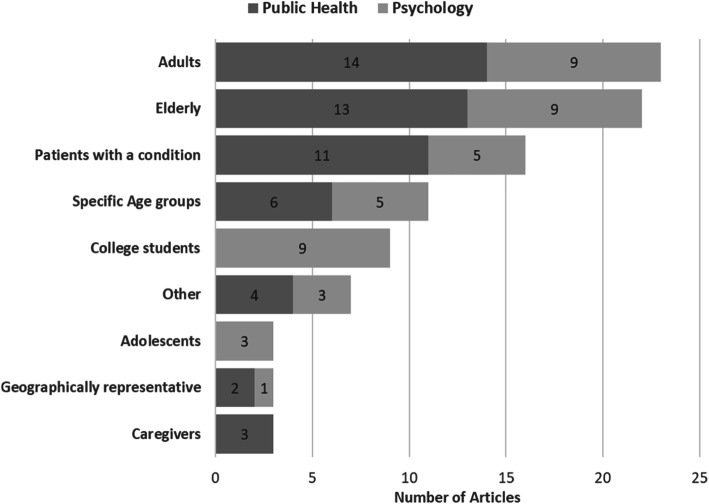
Distribution of studies by studied population

Next, we look at how the existing literature fits into this criterion. Figures 2 and 3 show the distribution of the reviewed studies across key areas, including study design, SWB type (EVA, EMO, or both), determinants and correlates, and study population.

#### Dimension of SWB studied

Of the 105 studies reviewed for determinants, only 36 looked at both EVA and EMO. The use of both dimensions of SWB is more prevalent in psychology (44% of the studies reviewed) than it is in public health (26% of the studies reviewed). Overall, the dominant SWB dimension studied was EVA, being featured in 42% of psychology studies and 65% of public health studies.

#### Measurement of SWB used

A common distinction when it comes to the use of measures of SWB is the use of single-item versus multi-item questions. For the studies reviewed, the use of multi-item questions was more common (90%) compared to single-item questions (14%). The distribution was relatively similar for studies from psychology and public health and across EVA and EMO. In the studies reviewed, 139 distinct measures were used to measure EMO and EVA. The most common measure of EVA was the Satisfaction With Life Scale (SWLS) and the most common measure of EMO was the Positive And Negative Affect Scale (PANAS). The SWLS was used in 22% of the studies in public health and 40% of the studies in psychology. Similarly, PANAS was used in 4% of the studies in public health and 18% of the studies in psychology.

#### Design of study

In terms of study design, 83 (a majority) of the studies were cross-sectional, 15 were longitudinal, and 7 employed both designs. Cross-sectional studies were more common in psychology (81%) than they were in public health (77%). None of the studies used an experimental or quasi-experimental design.

#### Study population

In terms of study populations (Fig. 3), 23 studies focused on adults; 22 focused on the elderly (ages 50 and above); 16 focused on patients with a medical condition such as obesity, mental illness, or cancer; 9 focused exclusively on college students; and only three utilized geographically representative samples. A focus on patients with a given condition was more prevalent in public health studies, while a focus on college students was limited to psychology studies.

Table 1 provides information on the 105 studies reviewed.

**Table 1:** Summary of reviewed studies

S. No.	Article	Discipline	Temporal coverage	Study population	SWB dimension	Determinants and correlates of SWB						
						Basic demographics	SES	Health and functioning	Personality	Social Support	Religion and culture	Geography and infrastructure
1	Abbott and Byrne 2012 [53]	PSY	CROSS	College students	EVA			D		X		
2	Abdel-Khalek 2011 [54]	PSY	CROSS	College students	EVA			X			D	
3	Allen et al. 2012 [55]	PSY	CROSS	Ages 63+	EVA			X	D			
4	Andrews 1986 [56]	PSY	CROSS	Multiple (Review)	EVA+EMO	X	X	X	X	X	X	X
5	Ardelt and Edwards 2015 [57]	PSY	CROSS	Ages 71–77	EVA	X	X	X	D	X		
6	Arent et al. 2000 [58]	PH	CROSS + LONG	Older adults (60+)	EMO			D				
7	Bradburn and Caplovitz 1965 [59]	PSY	CROSS	Adults	EMO	X	X	X				
8	Bradburn 1969 [60]	PSY	CROSS	Adults	EMO	X	X	X		X		
9	Braun 1978 [61]	PSY	CROSS	Ages 16–74	EVA+EMO	X	X	X			X	
10	Brdarić et al. 2015 [62]	PH	CROSS	Avg. age 28.93	EVA+EMO			D				
11	Calys-Tagoe et al. 2014 [63]	PH	CROSS	Ages 50+	EVA	D	X					
12	Campbell et al. 1976 [64]	PH	CROSS	Adults	EVA+EMO	X	X	X		X	X	
13	Campbell 1981 [65]	PH	LONG	Adults	EVA+EMO	X	X	X		X		X
14	Cantril 1965 [66]	PSY	CROSS	Multiple countries	EVA		X	X		X	X	X
15	Chen and Page 2016 [67]	PH	LONG	Ages 17–25	EVA	D	X					
16	Cheng 2004 [68]	PSY	CROSS	Adult	EVA+EMO	D	X	X		X		
17	Chou 1999 [69]	PSY	CROSS	Young adults	EMO	X				D		
18	Clemente and Sauer 1976 [70]	PH	CROSS	Adults	EVA	D	X	X		X	X	
19	Clemente and Sauer 1976 [71]	PH	CROSS	Adults	EVA	X	D	X			X	
20	Costa and McCrae 1980 [72]	PSY	CROSS + LONG	Adults	EVA+EMO				D			
21	Cramm et al. 2010 [73]	PSY	CROSS	Poor	EVA		D	X		X		
22	Cubí-Mollá et al. 2014 [74]	PH	CROSS	Parkinson’s patients	EVA	X	X	D				
23	Derdikman-Eiron et al. 2011 [75]	PSY	CROSS	Adolescents	EVA	D	X	X		X		
24	Deserno et al. 2017 [76]	PH	CROSS	Autism spectrum disorder patients	EVA	X		X		D		
25	Diener et al. 2003 [36]	PSY	CROSS	NA (Review)	EVA+EMO				X		D	
26	Dierk et al. 200 [77]	PSY	CROSS	Obese	EVA+EMO	X	X	D		X		
27	Edelstein et al. 2016 [78]	PH	CROSS	Glioblastoma patients	EMO			D				
28	Emerson and Hatton 200 [79]	PH	CROSS	Mentally disabled	EVA	X	X	D		X		
29	Fox 1999 [121	PH	CROSS	Multiple (Review)	EVA			D				
30	Freudiger 1983 [80]	PH	CROSS	Adult women	EVA	X	D	X			X	
31	Frey and Stutzer 2010 [81]	PSY	LONG	NA (Review)	EVA		D		X			
32	George and Landerman 1984 [82]	PH	CROSS + LONG	NA (Review)	EVA+EMO	X	X	X		X	X	
33	Grant et al. 2009 [83]	PSY	CROSS	Adults	EVA+EMO				D			
34	Guite et al. 2006 [84]	PH	CROSS	Adults	EVA	X	X					D
35	Gull and Dawood 2013 [85]	PH	CROSS	Elderly	EVA+EMO	X	X				D	
36	Hamilton et al. 2007 [86]	PSY	CROSS	Adults	EVA+EMO	X		D				
37	Hammond et al. 2014 [87]	PH	CROSS	Caregivers	EVA	X	D					
38	Hnilica 2011 [88]	PH	CROSS	Ages 15+	EVA+EMO		X			D		
39	Ickovics et al. 2006 [89]	PH	LONG	Female HIV patients	EMO	X	X	D				
40	Jia et al. 2017 [90]	PSY	CROSS	Migrant adolescents	EVA+EMO	X	X		D	D		
41	Jivraj and Nazroo 2014 [57]	PH	CROSS	Ages 50+	EVA	X	X	X				
42	Jivraj et al. 2014 [91]	PSY	LONG	Ages 50+	EVA	D	X	X		X		
43	Ju et al. 2013 [92]	PH	CROSS	Elderly women	EVA+EMO		X	X	D			
44	Kahneman and Deaton 2010 [93]	PH	CROSS	Adults	EVA+EMO	X	D	X			X	
45	Kaliterna Lipovčan et al. 2007 [94]	PH	CROSS	Adults	EVA+EMO		D					
46	Kasl and Harburg 1975 [95]	PH	CROSS	Adults	EMO	X	X					D
47	Khan and Husain 2010 [96]	PSY	CROSS	College students	EVA				D	X		
48	Kim et al. 2012 [97]	PSY	CROSS	College students	EVA+EMO			X	D	X		
49	Krause 2003 [98]	PSY	CROSS	Elderly	EVA	X	X		X		D	
50	Kunzmann et al. 2000 [99]	PSY	CROSS + LONG	Elderly	EMO	X		D				
51	Kutek et al. 2011 [100]	PH	CROSS	Rural men	EVA	X	X	D		X		
52	Lamu and Olsen 2016 [101]	PH	CROSS	Adults	EVA	X	X	X		X		
53	Lee and Browne 2008 [102]	PH	CROSS	Rural residents	EVA	X	X	D				
54	Lemola et al. 2013 [103]	PH	CROSS	General population	EVA+EMO	X	X	D				
55	Li and Fung 2014 [104]	PSY	CROSS	Married couples	EVA	X	X			X		
56	Li et al. 2016 [105]	PSY	CROSS	Adults	EVA+EMO	X	D					
57	Librán 2006 ]139]	PSY	CROSS	College students	EVA+EMO				D			
58	Linna et al. 2013 [106]	PH	CROSS	Young adult twins	EVA	X	X	D				
59	Liu et al. 2016 [107]	PH	CROSS	Ages 95+	EVA+EMO	X	X	D				
60	Lu et al. 2015 [108]	PH	CROSS	Adult caregivers	EVA	X	D	X				
61	Lu 2006 [109]	PSY	CROSS	College students	EVA	X					D	
62	Ludwig et al. 2012 [110]	PH	LONG	Program participants	EVA	X	X					D
63	Ma et al. 2015 [111]	PSY	CROSS	Adolescents	EVA	X			D			
64	Magallares et al. 2014 [112]	PSY	CROSS	Obese	EVA+EMO	X				X		
65	Malathi et al. 2000 [113]	PH	LONG	Staff	EVA			D		X		
66	McDonough et al. 2014 [114]	PSY	LONG	Breast cancer survivors	EVA	X	X	X		D		
67	Medley 1980 [115]	PH	CROSS	Adults (22+)	EVA	X	D	X				
68	Mhaoláin et al. 2012 [116]	PSY	CROSS	Elderly	EVA	X	X	D	X			
69	Michalos 1980 [22]	PH	CROSS	Adults	EVA	X	X	X	X	X		
70	Moskowitz et al. 2009 [10]	PSY	CROSS + LONG	Multiple (Review)	EMO			D				
71	Olsson et al. 2014 [117]	PH	CROSS	Ages 69–79	EVA	X		D				
72	Ozcakir et al. 2014 [118]	PH	CROSS	Patients	EVA	X	X	D				
73	Palmore 1979 [119]	PH	LONG	Older adults (60+)	EVA	X	X	D				
74	Pérez-Garín et al. 2015 [120]	PSY	CROSS	Mentally disabled	EVA+EMO			D				
75	Persoskie et al. 2014 [17]	PH	LONG	Adult patients	EVA+EMO	X	X	D	X			
76	Pinquart and Sörensen et al. 2000) [121]	PH	CROSS	Elderly	EVA	X	X	X		X		
77	Reis et al. 2013 [122]	PH	LONG	Adult patients	EVA+EMO			D				
78	Riddick 1980 [123]	PH	CROSS	Women (representative sample for the US)	EVA		D	X				
79	Rijken et al. 1995 [124]	PSY	CROSS	Elderly women	EVA			D	X			
80	Sand and Gruber 2018 [125]	PH	CROSS	Elderly	EMO	X	X	X				
81	Sandstrom and Dunn 2014) [126]	PSY	CROSS	College students	EVA+EMO				X	D		
82	Sharma et al. 2008 [127]	PSY	LONG	Adults	EVA			D				
83	Shiah et al. 2016 [128]	PH	CROSS	Adults	EVA						D	
84	Soto 2015 [129]	PSY	LONG	Nationally representative sample for Australia	EVA+EMO				D			
85	Spinhoven et al. 2015 [130]	PSY	LONG	Adults with an emotional disorder	EMO	X	X	X	D			
86	Spreitzer and Snyder 1974 [131]	PH	CROSS	Older Adults (65+)	EVA	X	X	X			X	
87	Steverink et al. 2001 [132]	PSY	CROSS	Ages 40–85	EVA+EMO	D	X	X		X		
88	Strobel et al. 2011 [133]	PSY	CROSS	College students	EVA				D			
89	Strózik et al. 2016 [134]	PSY	CROSS	Ages 8–12	EVA		X	X		X		X
90	Tanksale 2015 [135]	PSY	CROSS	Ages 30–40	EVA+EMO				D			
91	Taylor et al. 2000 [136]	PSY	CROSS + LONG	Multiple (Review)	EMO			X	D			
92	Tian 2016 [137]	PSY	CROSS	Elderly	EVA+EMO				X	D		
93	Uppal 2006 [138]	PH	CROSS	Disabled	EVA	X	X	D			X	
94	van Campen et al. 2013 [139]	PH	CROSS	Caregivers and non-caregivers	EVA	X	D	X				
95	Vos et al. 2010 [140]	PH	CROSS	Disabled	EVA	X		D				
96	Wadsworth and Pendergast 2014 [141]	PH	CROSS	Adults	EVA	X	X	D				X
97	Wang 2016 [142]	PSY	CROSS	Elderly	EVA+EMO					D		
98	Winstanley et al. 2015 [143]	PH	CROSS	Adults	EVA	X	X	D	X			
99	Wolinsky et al. 1985 [144]	PH	LONG	White elderly	EVA	X	X	X		X		
100	Wyller et al. 1997 [145]	PH	CROSS	Elderly	EVA	X		D				
101	You and Shin 2017 [146]	PH	CROSS	Middle-aged adults	EVA+EMO	X		D				
102	Yu and Chen 2016 [147]	PSY	CROSS	Adults	EVA+EMO	X	D	X				X
103	Yue et al. 2017 [148]	PSY	CROSS	College students	EVA	D			X			
104	Zank and Leipoid 2001 [149]	PH	CROSS	Dementia patients	EVA	X	X	D				
105	Zautra and Hempel 1984 [11]	PH	CROSS + LONG	NA (Review)	EVA+EMO			D				

Public health (PH); psychology (PSY); cross-sectional (CROSS); longitudinal (LONG); evaluation (EVA); emotions (EMO); evaluation and emotions (EVA+EMO); dominant or primary study determinant/correlate (D)

#### Determinants or correlates used

The seven broad categories of SWB determinants/correlates we identified are basic demographics, SES, health and functioning, personality, social support, religion and culture, and geography and infrastructure. Sixty-nine studies included basic demographics, 65 included SES, 74 included health and functioning, 27 incorporated personality, 35 included social support, 17 incorporated religion and culture, and only 9 included geography and infrastructure. A focus on basic demographics, SES, and health and function was more prevalent in public health studies, while a focus on personality was more prominent in psychology studies. Only 59 studies focused on three or more determinant/correlate categories.

In addition to the issues identified, when assessing existing studies based on our comparability criterion, we also found that of the studies reviewed only 21% included discussions of SWB theories (while not necessarily testing them). Inclusion of theories was more common in psychology (27%) than in public health (17%) and more common when exploring EMO (28%) than EVA (17%).

To better understand if and how information from existing studies can be used to test SWB theories and improved to encourage comparability, we delve deeper into how these determinants and correlates influence the two SWB outcomes of EVA and EMO. One thing we would like to make clear is the distinction between determinants and correlates of SWB. The term determinant has the implicit connotation of causality which may not necessarily be the case between all variables and SWB. While this study was initially aimed at looking at determinants of SWB, a lack of longitudinal research which aids in such causal inferences led us to realize that what studies often referred to as determinants of SWB were at best correlates.

#### Basic demographics

Basic demographics include age, gender, and race/ethnicity. For the studies reviewed, we found that basic demographics were mostly used as control variables. In studies explicitly focused on basic demographics (indicated by a “D” under the “determinants and correlates” columns in Table 1), age was investigated the most, followed by gender and race/ethnicity. We found that age- and race/ethnicity-centered studies tended to focus more on EVA, while gender-focused studies were more concerned with EMO.

Studies of the direct influence of age on SWB have typically focused on EVA, and they were found to have inconsistent results. Some studies showed a U-shaped relationship, typically flattened at age 40 [88, 138], while others showed a positive association [66, 71, 118, 139, 150], a negative association [59, 63, 101, 138], or no association [56, 100, 112, 117]. While these studies relied on absolute age, other studies focused on the process of aging and suggested that changes in socioeconomic circumstances, personality, and health related to age are what truly influence SWB [67, 99, 132, 134]. For instance, in the elderly, aging has been associated with physical decline, continuous personal development, change in familial responsibilities, retirement, and social loss, all of which have been found to influence SWB [57, 68, 92, 103, 132, 137].

Gender-focused studies have also shown mixed results; for example, being female has been found to be both positively [101, 102, 118, 132] and negatively [63, 107, 132] associated with SWB. General agreement, however, surrounds the idea that women are more susceptible to intense affective responses because they report greater negative and positive emotions compared to men in similar situations [61, 69, 151]. Gender differences in certain personal characteristics—such as self-efficacy, self-esteem, gratitude, optimism, and propensity to conditions such as depression and anxiety—have also been shown to create discrepancies in the emotions experienced [75, 111, 148]. Limited research in the United States (US) has suggested that minorities, such as African American, Asian, and Hispanic women, report lower SWBs than do their White counterparts [56, 60, 141]. Overall, discrepancies in findings about how basic demographics influence SWB may be attributable to the fact that SES-, health-, and personality-related factors are inconsistently controlled for in these studies [67, 99, 132].

#### SES

SES includes income, education, employment, immigration status, and family structure. Similar to basic demographics, we found that most studies used some SES correlates as control variables. Within SES-focused studies, income received the most attention, followed by family structure, employment, education, and immigration status. Measures of SES tended to be objective in nature, such as income, being married, and being employed; however, in many cases, subjective measures, such as wealth satisfaction, income adequacy, and satisfaction with marriage, were also used. In general, SES studies focused more on EVA compared to EMO. Of all SES determinants/correlates, family structure–related studies paid the most attention to EMO.

There is a consensus that income positively influences SWB [73, 94]. How it does so, however, is constantly debated. Some suggest that higher income may not necessarily increase SWB but does buffer the impacts of negative emotions, such as worry [105, 147, 152]. Income may also exert a greater influence on SWB at extreme levels of poverty, but once basic needs are met, the influence wanes [93]. Income can also relate to specific aspects of life that may lead to higher EVA, such as satisfaction with material status, social status, health, achievement, and future security [82, 94, 105, 121, 144]. Education has been found to be weakly related to EVA [101, 118, 119], and this relationship is influenced significantly by income and other socioeconomic variables [59, 63, 73, 103, 118, 131, 150]. Unemployment has been found to have a detrimental impact on SWB that extends beyond the obvious financial difficulties to include loss of self-esteem, social stigma, stress, anxiety, unhealthy behaviors, and other health issues [64, 81, 101, 102, 138, 141, 153].

Limited research also suggests that immigrants typically have lower SWB than do less recently settled residents, which can be attributed to assimilation needs and the availability of resources, but these differences decrease with time of residence [125, 138]. In terms of family structure, being married or living with a partner—and being satisfied with the relationship—have a positive influence on SWB [22, 64, 80, 82, 88, 101, 116, 144, 150]. However, the influence varies based on age and gender [104]. Studies on having children are generally limited and inconclusive [56, 82], with a few studies finding that living with children may lead to higher EVA and negative emotions [132, 147]. Informal caregiving for children, parents, relatives, or the disabled has a negative impact on SWB if the care-receiver is disabled or if the caregiver has a full-time job in addition to having caregiver responsibilities [87, 139]. A combination of family structure and income satisfaction has also been found to influence emotions in terms of what one worries about and how one thinks these worries can be resolved [61, 66, 82, 88, 94, 105].

The combination of determinants/correlates that constitute SES also influence EVA by determining an individual’s standard of comparison and the resources available to cope with adversity in life [81].

#### Health and functioning

Health and functioning include general health status, body weight, physical activity, sleep, disability, and specific diseases. Across all studies reviewed, the use of general or self-reported health as a control variable was very common. Within health and functioning-focused studies, specific diseases received the most attention, followed by general health, physical activity, disability, body weight, and sleep. Measures of health and functioning tended to include both objective and subjective measures. The most common subjective measure was general or self-reported health, followed by perceived intrusiveness of any health condition and perceived physical and mental functioning. Once again, we found these studies to have an EVA focus, with some attention being paid to EMO. Studies related to specific diseases and physical activity typically paid more attention to EMO.

There is a consensus that general health status and self-reported health are positively and strongly associated with EVA, even after controlling for other determinants/correlates [11, 82, 101, 102, 123]. The perception of intrusiveness in terms of reduced cognitive and physical functioning influences this association [78, 154]. Developmental disabilities, physical disabilities, and mental disorders (e.g., bipolar, depression, or anxiety) all influence SWB primarily through the impairments they cause to functioning [75, 76, 107, 118, 130, 140, 149, 155]. Specific medical conditions and diseases, such as pre-term birth, HIV-positive status, and cancer, have negative impacts on SWB [10, 89, 143]. Insomnia, day-to-day variability in sleep, subjective sleep quality, and average sleep duration have all been found to influence EVA [86, 103, 156–158]. The increased severity of all the conditions mentioned above has also been consistently associated with lower SWB [53, 74, 86, 138, 159, 160]. Such conditions cause physical, psychological, economic, and social suffering, which impact SWB [122, 161]. Body weight, in terms of high and low body mass index (BMI), lowers SWB [106], with women reporting more significant effects from weight-related issues on SWB, in general [77, 112]. Physical activity has consistently been found to influence EVA and EMO positively [58, 146, 160] through its direct effect on physical health and its ability to counteract depression, stress, and anxiety [95, 113, 127, 146].

In addition to their direct effects on EVA and EMO, health and function determinants/correlates can also have indirect effects through their influence on other aspects of life, such as personal control, social engagement, social satisfaction, discrimination, stigma, marriage, employment, income, independence, and self-esteem [62, 76, 77, 79, 112, 120, 140, 141]. Gender, SES, culture, social support, personality, and differences in resource availability have also been found to influence the relationship between health and functioning and SWB [11, 17, 75, 79, 114, 136].

#### Personality

Studies on personality fell into one of two distinct groups: those that looked at the Big Five personality traits (i.e., extroversion, agreeableness, openness, conscientiousness, and neuroticism) and those that investigated more nuanced personality attributes (e.g., optimism, self-esteem, and self-efficacy). Both groups tended to be well researched, and equal attention was paid to EVA and EMO. Personality was typically measured using subjective personality scales.

There seems to be a consensus on how the Big Five and more nuanced personality attributes influence SWB. For the Big Five, conscientiousness is positively associated with EVA, extroversion is associated with positive emotions, and neuroticism is associated with negative emotions [72, 83, 135, 162]. Extraverted individuals tend to have a stronger emotional reaction to positive events, while neurotic people tend to have stronger reactions to negative events [129]. More nuanced personality traits, such as self-efficacy, self-esteem, and optimism, also mediate the effect of personality on EVA [96, 133]. Adaptation has been found to influence both EVA and EMO [72, 162]. Some studies have also found evidence of a reciprocal relationship between SWB and personality traits, in which people who are initially extraverted, agreeable, conscientious, and emotionally stable report higher EVA, and people with higher EVA become more extraverted, agreeable, conscientious, and emotionally stable over time [129].

#### Social support

Studies in this category tended to measure social support using both objective means (e.g., number of social contacts or frequency of interaction) and subjective means (e.g., quality of social contacts or satisfaction with social contacts). Equal attention was paid in such studies to EVA and EMO aspects of SWB.

Studies have consistently found that perceived social support from family, community, and friends and acquaintances yielded a positive effect on SWB [73, 96, 100]. Studies indicate that the size of the social network [117], quality of relationships [121, 126], and interaction frequency [69] all influence EVA. Moreover, a lack of social support and discrimination (e.g., based on age, gender, or immigration status) exerts downward pressure on EMO [88, 90]. These findings also identify potential mediators between social support and SWB, such as loneliness, self-esteem, and stress [100, 137]. Social support may also be more critical to EVA for the elderly or for individuals with health challenges or disabilities as compared to the general population [69, 114, 121, 132].

#### Religion and culture

Religion and culture receive equal attention in the literature reviewed for this study, and the two determinates often tend to be intertwined. They are typically measured using both subjective means (e.g., religiosity or individualist vs. collective culture) and objective means (e.g., number of visits to a place of worship). These studies tended to focus on EVA. Due to the limited number of studies in this category, it may be premature to make claims regarding the consistency of the findings.

Current research suggests that religion and culture impact SWB through a variety of pathways, including psychological ramifications, coping mechanisms, and conceptualization of SWB. Studies on religion tended to focus on non-US populations and rarely controlled for socio-demographic variables. Ellison [163] suggested that religion may yield psychological benefits that result in better SWB, such as helping individuals deal with and resolve problematic situations, and supporting self-esteem and self-efficacy. EVA is most often found to be positively associated with religiosity (defined as identifying as being actively religious) and frequent visits to places of worship [54, 85, 98]. Religion and culture also impact EVA through their influence on how SWB is conceptualized (e.g., if is it an individual or collective concept), how comparison standards are formulated for EVA, and optimism [66, 109, 128]. Religions and cultures also influence EMO through optimism, coping with life events and stress, and how individuals feel they fit within the larger cultural context [98, 128].

#### Geography and infrastructure

Studies in this category tended to rely on both objective means (e.g., physical location or economic conditions) and subjective means (e.g., perception of access) to measure geography and infrastructure. The primary focus was EVA. Once again, due to the limited number of studies in this category, it may be premature to make claims regarding the consistency of the findings.

The studies focused on EVA and tended to be comparative by nature, looking at different neighborhoods or communities and comparing the EVA of their inhabitants. Lower EVA has been reported for urban residents compared to rural residents [134] and for residents living in economically disadvantaged areas [110]. EVA is also influenced by location-based (e.g., country-specific or state-specific) life priorities and comparison standards that lead to certain aspirations and worries [66]. Limited studies also indicate that access disparities for basic services, such as food, water, and sanitation; living conditions in the home; and opportunities for recreation and transportation can influence SWB [84, 95, 110].

Due to our focus on public health and psychology, studies from other disciplines related to geography and infrastructure were not included in this review. However, it is of value to highlight some findings from other disciplines to identify potential research directions for public health and psychology. In terms of geography, studies have found that distinct SWB determinants/correlates arise at multiple geographical scales. At the national level, Veenhoven and Ehrhardt’s [164] livability theory suggests that certain characteristics exist across cultures that make the quality of life in some countries higher than it is in others. This is supported by research that suggest that the nations with the highest SWB tend to experience economic development and wealth; a strong rule of law and human rights; lower corruption; effective and efficient governments; progressive taxation laws; income and job security programs; political freedoms and protection; lower levels of unemployment; better overall health; and income equality [165]. At the regional level, those in urban areas (larger counties and metropolitan areas in particular) have been reported to have lower EVA due to higher pollution, traffic, crime, living costs, congestion, and alienation, and a lack of green spaces [166]. Similarly, local labor market conditions, the local price of goods and services, and regional amenities have been found to influence SWB [166, 167].

Studies related to infrastructure can be combined with studies looking at city/neighborhood level determinants/correlates of SWB, as the geographic scale of these studies typically overlaps [167, 168]. At the city/neighborhood level, parks, safety, amenities (such as grocery stores and cultural facilities), social support (such as interacting with neighbors), economic features (such as the cost of living and commuting times), institutional features (such as the quality of government services), and environmental conditions (such as pollution) have all been linked to SWB [167, 168]. Peoples’ subjective appraisals of the environment in which they live have also been found to impact SWB [169]. Finally, numerous aspects related to the natural environment at multiple geographical scales—such as access to natural spaces, panoramas and landscapes, biodiversity, lower air and noise pollution levels, cultural and recreational value, health-related services, and aesthetic experiences—have all been linked to higher SWB [154, 165, 168, 170]. Conversely, climate change and the degradation of nature have negative effects on SWB at both the local and global scale [171]. With climate change comes biodiversity loss, food contamination, invasive species, and environmental pollution, which are all associated with lower SWB [171, 172].

Overall, the studies reviewed in this section highlighted the potential determinants/correlates of SWB and how they might interact with one another. Given that most of these studies included a limited number of determinants/correlates and control variables—only 59 of 105 studies focused on 3 or more determinant categories—the findings lack consistency, and opportunities for comparisons are rare. This issue also brings into question the relatively consistent findings regarding how SWB is influenced by income, general health, disability, physical activity, personality, and social support. In addition, the pathways through which these determinants/correlates influence SWB remain insufficiently researched.

Based on our review, we find that the seven categories of determinants/correlates of SWB can potentially be used to improve the link between theory and empirical research and that the overlap in the categories as they relate to our four theory categories enables us to conduct theoretical testing across theories in unison. While given the lack of systematic reviews at this point it is premature to suggest conceptual frameworks, we can still highlight how theory and empirical literature potentially connect.

##### Fulfillment and engagement theories

Basic demographics, SES, and personality play a role in goal and need formulation, engagement in various activities, and the resources available (personal, social or monetary) to meet goals and needs. In addition, personality influences how people adapt to their situations and cope with goal and need fulfillment or lack thereof. Health can significantly influence need and goal fulfillment by enabling or restricting activities while social support can act as a resource and coping mechanism. Religion and culture can shape need and goal formulation, influence SWB consequences of meeting or not meeting them, impact the value associated with conducting activities, and be a resource for coping with life events and stress. Finally, geography and infrastructure influences access to activity opportunities and resources to meet goals and needs.

##### Personal orientation theories

Basic demographics can influence personal orientation. In particular, the process of aging and gender can influence more nuanced personality traits such as optimism and self-efficacy. SES and social support can act as a resource and coping mechanism influencing more nuanced personality traits such as self-esteem. Aspects of health and functioning such as disability and the intrusiveness of medical conditions can also influence personal orientation. Religion and culture can influence personal orientation through their impact on long-term self-esteem, self-efficacy, and optimism. Finally, by promoting beneficial behaviors such as physical activity and access to beneficial settings such as nature, geography and infrastructure also influence personal orientation.

##### Evaluative theories

Basic demographics, SES, personality, health and functioning, religion and culture, and geography and infrastructure impact evaluation by playing a role in influencing standards of comparisons a person uses. SES also provides one with resources to cope with difficulties in life. Personality plays a role in determining whether people compare themselves to higher or lower standards and how they process the results of these comparisons. Social support impacts evaluation by acting as a resource and coping mechanism. Religion and culture also determine what is valued in the process of comparison as well as how people cope with results of the comparison. Geography and infrastructure influences who people compare themselves with in terms of proximity.

##### Emotion theories

Basic demographics such as age and gender influence the propensity to experience positive emotions while SES and health and functioning influence issues and worries one faces in life that determine the opportunities one has to experience positive emotions. Personality plays an important role in how we experience life events and experiences (e.g., optimists vs. pessimists) and how we adapt to these emotions. Social support is an important resource and coping mechanism for various life events. Religion and culture influence values systems that determine if we see experiences as positive or negative as well as provide support to cope with stressful events. Finally, geography and infrastructure influences localized experiences and stressors that influence emotions (e.g., crime, economy).

While initial, these findings suggest the potential of a comprehensive set of determinants or correlates to test theories. Systematic reviews in the future can help provide more evidence for these potential connections and aid in the creation of more holistic frameworks on the drivers of SWB.

## Discussion

Although we began with our review efforts with intent to develop a theoretical and methodological framework that would make empirical research comparable in a systematic way, our efforts end up providing more value in revealing the ways that SWB literature lacks a coherent theoretical and methodological framework than attempting to develop such a framework. In our review, we found that existing theories on SWB can be both contradictory and overlapping and that there is a need to explore connections between theories by developing unified propositions that can be tested. Further, there is a lack of a clear, unifying theoretical basis for categorizing and comparing empirical studies. As a result, we did not formulate a theoretically informed criterion to group existing empirical literature, but rather operationalized the organization of empirical literature based upon the dimension of SWB studied, measure of SWB used, design of the study, study population, and types of determinants and correlates to organize and summarize the empirical literature. Based on our review of empirical literature on SWB, we found that the seven categories of determinants/correlates identified can potentially be used to improve the link between theory and empirical research and that the overlap in the determinant/correlates as they relate to multiple theory categories enables us to conduct testing of theories in unison. However, to do so in the future would require a conscious effort by researchers in several areas.

First, this review identified several determinants and correlates that have not received adequate attention in existing research, including race/ethnicity, education, immigration status, religion and culture, and geography and infrastructure. Among the public health studies reviewed, 15% focused on a single determinant category, and 67% included three or more determinant categories. Among the psychology studies reviewed, 20% focused on a single determinant category, and only 42% included three or more determinant categories. Even when similar categories were used, different determinants/correlates within them were analyzed. For instance, for SES, some studies evaluated income while others evaluated income adequacy, or, for social support, some studies used the number of social contacts while others used the perceived quality of social contacts. We also included a few studies on geography and infrastructure from disciplines other than public health and psychology and found that these studies highlighted the fact that understanding the spatial nature of SWB is important because peoples’ needs, desires, and potential methods to improve their SWB are all context sensitive. Therefore, future studies in public health and psychology should build on and further such research. Several inter-determinant relationships are suggested in this review, such as disability with social networks; age with physical decline, social loss, and personal development; and gender with self-efficacy, which are critical to understanding SWB and cannot be realized until holistic frameworks of determinants/correlates are tested. Future studies should focus on using a comprehensive set of SWB determinants/correlates to increase comparability across studies, enable researchers to progress from discovering SWB correlates to exploring effect sizes, and facilitate empirically testing theories. There is also a need for more comparative methods of analysis. In all the studies reviewed for this paper, no single prevalent analysis method in either discipline or across disciplines emerged; methods ranged from descriptive and simple correlation to structural equation modeling, confounding comparisons between them.

Second, there is a need to focus on data collection. The availability of data related to SWB is a constant problem and often a deterrent for research, especially at the local level; most studies, such as the World Values Survey and the Gallup World Poll, focus on macro-level analyses. The use of the macro datasets is restrictive, as researchers cannot control for variables which hampers comparability across datasets. Moreover, most of the reviewed studies relied on secondary data, and studies that collected original data contained homogeneous samples. For example, nine psychological studies focused just on data from college students, while only three—two from public health and one from psychology—used a geographically representative sample. Other homogenous samples were based on age, obesity, disease, or disability. While these studies are extremely valuable for specific demographics, the findings are inherently non-generalizable and non-comparable. The associated costs of primary data collection are also restrictive for academic researchers. However, to advance SWB research, more sophisticated, SWB-focused data needs to be collected, as opposed to relying on existing datasets.

Another issue related to reliance on secondary data is the inconsistency in the SWB dimension studied and use of SWB measures. Of the 105 studies reviewed for determinants, only 36 looked at both EVA and EMO. The use of both dimensions of SWB is more prevalent in psychology (44% of the studies reviewed) than it is in public health (26% of the studies reviewed). Overall, the dominant SWB dimension studied was EVA, being featured in 42% of psychology studies and 65% of public health studies. This EVA focus leads to a lack of research that looks at how EMO is influenced by determinants such as age, race/ethnicity, income, education, employment, immigration status, general health, body weight, disability, sleep, religion and culture, and geography and infrastructure. EVA measures are typically much simpler to collect, which may account for this research focus; however, favoring one aspect of SWB can be problematic. For instance, Morrison [173] indicated that place of residence would affect people’s judgments of EMO more than of EVA. Looking only at one or the other may overestimate or underestimate the effects of different SWB determinants and limits comparability of empirical research. Therefore, there is a need for more studies that incorporate both the EVA and EMO to determine factors influencing SWB more accurately Moreover, even when looking at similar dimensions of SWB, we found a significant amount of variation in the measures used. The most commonly used measures were the Satisfaction with Life Scale for EVA, and the Positive and Negative Affect Schedule for EMO. While we do not prescribe the use of any one measure for either, the use of so many different measures also impedes comparisons among these studies.

Finally, the current state of data collection does not lend itself to longitudinal research, as very few longitudinal surveys collect SWB data. Of the studies reviewed for this paper, only 23% of public health studies and 19% of psychology studies were longitudinal. More longitudinal studies are needed to elucidate the direction, effect size, and mediating or moderating relationships between SWB and its determinants. This type of research would also allow for the exploration of adaptation and SWB, and from the perspective of aging and lifecycle-based research in a rapidly changing world, longitudinal research might tease out temporal changes in SWB. Also, longitudinal data collection would enable the conducting of experimental research to facilitate interventions to improve SWB. As pointed out by other researchers [3] and evident in our review of empirical research, there seems to be adequate research on the correlates of SWB. What is needed now are longitudinal studies focused on assessing causality to identify determinants of SWB.

In progressing the study of SWB, it is necessary to understand how each field assesses and measures it and how research can be categorized and compared across disciplines in a systematic manner to build a better understanding of SWB over time. Defining a set of determinants for SWB creates the opportunity for a standardized study protocol in this emerging field. While we feel that the recommendations discussed above would help conducting studies that are more comparable in the future, for those pursuing more immediate systematic reviews of SWB determinants/correlates, we recommend assessing studies for inclusion based on the criterion discussed in this paper. This review examines SWB literature in psychology and public health, and future studies should consider how other fields are assessing SWB to yield a more thorough evaluation of both the state of SWB and its future directions. While this study begins to unpack these intricacies, continuing this methodology will ensure SWB and its determinants and correlates are appropriately and comprehensively delineated.

It is important to acknowledge the limitations of this review. The first limitation is its focus on only two disciplines. Much of the use of SWB within public health stems from psychological theories, and this degree of overlap provided a logical pairing. The two disciplines also presented a wealth of literature on determinants and correlates. However, we do intend to add more disciplines in future research. Another limitation of this review is that it is heavily focused on individual SWB at the expense of macro-societal forces that could be affecting SWB in entire communities. This focus leads to highly resource-intensive, individualized interventions, such as prescribing a specialized diet to reduce an individual’s BMI versus broader interventions that could improve SWB for a larger percentage of the population, such as revising policies to allow for urban agriculture. This focus on the individual can also lead to an ecological fallacy, wherein upon finding a relationship between individual SWB and individual BMI, one assumes a similar relationship between community SWB and community BMI. These limitations percolate through much of the literature base and thus must be accounted for when interpreting the findings. Finally, due to concerns of brevity, in this review we explicitly focus on empirical literature on how determinants and correlates influence EVA and EMO. Literature on the relationships between EVA and EMO, between determinants/correlates and specific theories (however rare), while equally important, were not explored in detail. Using the categories described in this study to systematically compare SWB studies, we hope to do this in the future.

## Data Availability

Not applicable.

## Abbreviations

SWB: Subjective well-being
EMO: Emotional/affective dimension of SWB
EVA: Evaluative/cognitive dimension of SWB
SES: Socioeconomic status
BMI: Body mass index
PANAS: Positive and Negative Affect Schedule
PH: Public health
PSY: Psychology
CROSS: Cross-sectional
LONG: Longitudinal
EVA+EMO: Evaluation and emotions
D: Dominant or primary study determinant
